# Intronic variant in *POU1F1* associated with canine pituitary dwarfism

**DOI:** 10.1007/s00439-021-02259-2

**Published:** 2021-02-06

**Authors:** Kaisa Kyöstilä, Julia E. Niskanen, Meharji Arumilli, Jonas Donner, Marjo K. Hytönen, Hannes Lohi

**Affiliations:** 1 Department of Medical and Clinical Genetics, University of Helsinki, Helsinki, Finland; 2 Department of Veterinary Biosciences, University of Helsinki, Helsinki, Finland; 3 Folkhälsan Research Center, Helsinki, Finland; 4 Genoscoper Laboratories Ltd (Wisdom Health), Helsinki, Finland

## Abstract

The anterior pituitary gland secretes several endocrine hormones, essential for growth, reproduction and other basic physiological functions. Abnormal development or function of the pituitary gland leads to isolated or combined pituitary hormone deficiency (CPHD). At least 30 genes have been associated with human CPHD, including many transcription factors, such as *POU1F1*. CPHD occurs spontaneously also in mice and dogs. Two affected breeds have been reported in dogs: German Shepherds with a splice defect in the *LHX3* gene and Karelian Bear Dogs (KBD) with an unknown genetic cause. We obtained samples from five KBDs presenting dwarfism and abnormal coats. A combined analysis of genome-wide association and next-generation sequencing mapped the disease to a region in chromosome 31 and identified a homozygous intronic variant in the fourth exon of the *POU1F1* gene in the affected dogs. The identified variant, c.605-3C>A, resided in the splice region and was predicted to affect splicing. The variant's screening in three new prospective cases, related breeds, and ~ 8000 dogs from 207 breeds indicated complete segregation in KBDs with a carrier frequency of 8%, and high breed-specificity as carriers were found at a low frequency only in Lapponian Herders, a related breed. Our study establishes a novel canine model for CPHD with a candidate *POU1F1* defect.

**Supplementary Information:**

The online version contains supplementary material available at 10.1007/s00439-021-02259-2.

## Introduction

The anterior pituitary gland is a critical endocrine organ composed of at least five differentiated cell types and responsible for the secretion of thyroid-stimulating hormone (TSH), growth hormone (GH), prolactin (PRL), adrenocorticotropin (ACTH), follicle-stimulating hormone (FSH) and luteinising hormone (LH) (Kerr et al. 2008; de Moraes et al. 2012). Abnormal development or function of the anterior pituitary gland leads to pituitary hormone deficiency either in an isolated or combined form. Combined pituitary hormone deficiency (CPHD) is characterised by a lack of growth hormone (GH) and at least one additional pituitary hormone. In isolated GH deficiency, the patient is deficient only for GH.

The genetics of CPHD involves at least 30 genes required in the anterior pituitary development, differentiation, and maintenance (Fang et al. 2016; Castinetti et al. 2016), including well-established transcription factor genes *PROP1* (Wu et al. 1998), *POU1F1* (Tatsumi et al. 1992), *HESX1* (Dattani et al. 1998), *LHX3* (Netchine et al. 2000), and *LHX4* (Machinis et al. 2001). CPHD prevalence is 1:8000, and cases can be categorised into syndromic and non-syndromic groups according to the molecular defects (De Rienzo et al. 2015). A recent genotype screening of the known CPHD genes in ~ 1200 patients revealed only 7.3% and 29.5% discovery rates in sporadic and familial cases, respectively (Jullien et al. 2020). Indeed, the molecular aetiology remains unexplained in most patients despite the accelerating speed of gene discoveries facilitated by high throughput sequencing. Further research is warranted to improve early diagnostics and care since hormone deficiency at birth can result in hypoglycemia and death.

CPHD is spontaneously present also in animals, including mice and dogs. The Snell and Jackson dwarf mice have defective Pou1f1 and lack thyrotroph, somatotroph, and lactotroph development (Bodner et al. 1988; Li et al. 1990). The Ames dwarf mouse with a Prop1 mutation manifests a broader phenotype than the Snell and Jackson mice with an additional lack of gonadotropin levels (LH and FSH) (Sornson et al. 1996). Mouse models have played a fundamental role in understanding pituitary development, function, and disease (Castinetti et al. 2016).

In dogs, pituitary dwarfism has been reported in German Shepherds (Voorbij et al. 2014) and Karelian Bear Dogs (KBD) (Andresen and Willeberg 1976). The affected German Shepherds have a proportionally small stature, a puppy-like (lanugo) coat that lacks most primary or guard hairs, symmetrical alopecia, and a combined deficiency of GH, TSH, and PRL (Allan et al. 1978; Kooistra et al. 2000). The condition is caused by an autosomal recessive 7-bp intronic deletion in *LHX3*, resulting in defective splicing (Voorbij et al. 2011). The same variant is also present in the related Saarloos and Czechoslovakian Wolfdogs breeds (Voorbij et al. 2014).

Similar to the affected German Shepherds, the affected KBDs also present with growth retardation and coat abnormalities. Andresen and Willeberg (1976) described nine dwarf KBDs from six litters that were approximately 15–20 cm smaller than unaffected KBDs. Some of the affected animals retained their puppy coat, whereas some had a regular appearing adult coat before losing most of it at 2–3 years. Two affected dogs had low somatomedin levels. Pedigree analysis suggested an autosomal recessive disease (Andresen and Willeberg 1976). After 45 years since the original description of the KBD phenotype, we have now identified similar cases from Finland and Sweden and uncovered a candidate causative variant in the *POU1F1* gene.

## Materials and methods

### Study cohorts, pedigree and ethics approval

Canine DNA samples were retrieved from the canine DNA bank at the University of Helsinki, including the KBD sample cohort of 642 dogs utilised in variant discovery and validation. In addition, 263 Lapponian Herders and 87 Laika dogs were used in variant validation. Furthermore, *POU1F1* genotype data from 7925 dogs submitted for commercial testing was obtained from Genoscoper Laboratories (Wisdom Health, Helsinki, Finland). We received pedigree information for the affected KBDs from the Finnish Kennel Club’s pedigree registry KoiraNet and a pedigree was drawn using the GenoPro genealogy software. All sampled animals were privately owned pet dogs enrolled in the study with the owner’s informed consent. Sample collection was ethically approved by the Animal Ethics Committee of State Provincial Office of Southern Finland (ESAVI/343/04.10.07/2016 and ESAVI/25696/2020).

### Association study

To conduct genome-wide association analysis (GWAS), five affected and 139 unaffected KBDs were array genotyped in four batches with the Illumina CanineHD Beadchip containing 172,963 markers. Before analysis, quality control was performed to remove samples with < 93% call rate and markers with < 95% call rate, < 5% minor allele frequency (MAF), or Hardy–Weinberg equilibrium (HWE) *p* value < 1 × 10^–7^ in control dogs. Pruning for call rate was performed before merging of batches and for MAF and HWE after merging. After QC, 144 dogs and 96,274 markers remained. GWAS was performed with plink v1.90b6.10 (Purcell et al. 2007) using basic case–control association analysis (--assoc). *P* values were corrected for inflation with genomic control correction and for multiple testing with the Bonferroni method. Stratification was evaluated from quantile–quantile and multidimensional scaling plots after analysis. Multidimensional scaling was performed with plink using commands –cluster and –mdsplot 2. Results were visualised using python’s matplotlib module (Hunter 2007).

### Next generation sequencing and bioinformatics

Whole genome sequencing (WGS) data from three affected KBDs was produced. Using a 350 bp insert size PCR-free DNA library preparation method, two affected dogs were whole-genome sequenced at 26× and 30× coverage on Illumina high-throughput sequencing platform with a read length of 300 bp (paired-end reads, 2 × 150 bp) at the Novogene Bioinformatics Institute (Beijing, China). Another affected dog was whole-genome sequenced at 14× coverage on HiSeq Illumina sequencing platform with a read length of 252 bp (paired-end reads, 2 × 126 bp) at the University of Bern. The three WGS samples were submitted to the Sequence Read Archive in BAM format with BioProject accession number PRJNA684429 and BioSample accession numbers SAMN17057666, SAMN17057667, and SAMN17057668. The entire next-generation sequencing (NGS) cohort comprised 1038 animals (Online material 1, Suppl Table 1), including WGS data from 804 control animals and whole exome sequence (WES) data from 231 animals. The control cohort was provided by the Dog Biomedical Variant Database Consortium (Jagannathan et al. 2019), and a subset was obtained from the dog and wolf genome SNP database (DoGSD) (Bai et al. 2015).

The reads from the sequenced samples were mapped to the dog reference genome canFam3.1 as described previously (Hytönen et al. 2016). Single nucleotide variant calling was performed as described previously (Hytönen et al. 2016). In addition, mobile-element insertions were detected by MELT (Gardner et al. 2017), and structural variants including deletions, duplications, inversions and insertions by DELLY2 (Rausch et al. 2012). Functional effects of the variants were predicted using ANNOVAR with Ensembl release-100 and NCBI Canis lupus familiaris Annotation Release 105 annotations. The filtering of the single nucleotide variants and small insertions and deletions was performed with the command line version of GQT (Layer et al. 2016), assuming autosomal recessive inheritance. Thus, the affected dogs (case 1 and 4) were required to share the variants in a homozygous state, and controls were allowed to be heterozygous, wild-type, or uncalled. The filtering of the mobile element insertions (MEIs) and structural variants (SVs) was performed using the webGQT variant analysis interface (Arumilli et al. 2020). The analysis was performed with a subset of the whole genome samples under the assumption of autosomal recessive inheritance and with 10 heterozygous animals allowed for the alternative alleles in the control set. All genetic analyses were performed using the dog genome build CanFam 3.1.

Bioinformatic analyses were performed on the prioritised sequence variants obtained from NGS data analysis. Pathogenicity of the missense variants was estimated using the PredictSNP (https://loschmidt.chemi.muni.cz/predictsnp1/) tool, which provides a consensus in silico prediction from several different prediction algorithms (Bendl et al. 2014). The splice region's possible splice effect and synonymous variants were predicted with the NNSPLICE 0.9 tool (Reese et al. 1997). Conservation of the indel and missense variants' positions was assessed using the Multiz 100 vertebrate alignment available in the UCSC Genome Browser. The evolutionary conservation of the *POU1F1* splice region variant position was determined by constructing multiple sequence alignment with the Clustal Omega algorithm (https://www.ebi.ac.uk/Tools/msa/clustalo/). The aligned sequences were retrieved from the Entrez protein database (https://www.ncbi.nlm.nih.gov/protein/).

### Variant validation

The identified *POU1F1* variant was confirmed and validated with Sanger sequencing and Taqman genotyping. Primer3 (http://bioinfo.ut.ee/primer3/) (Rozen and Skaletsky. 2000) was used to design the following Sanger sequencing primers; 5′-CCAGGAAAAGTGTGATCGGG-3′ (forward) and 5′-TCCATCTCCTCTGTACGTTTTG-3′ (reverse). Biotools DNA Polymerase (Biotools B&M Labs, S.A.) was used to perform PCR amplification, Sanger sequencing reactions were performed at the Institute for Molecular Medicine Finland (FIMM), and the obtained Sanger sequence data were analysed with the Sequencher program (Gene Codes Corporation). After initial Sanger confirmation, a custom Taqman SNP genotyping assay (ThermoFisher Scientific) was ordered to genotype the *POU1F1* variant in larger sample cohorts. The primer and probe sequences for the assay were the following: 5′-TTTGCATTGTTTTAGAAAGAAAATTTGAAACTCAAA-3′ and 5′-CCTTTTTCTTTCATTTGCTCCCACTT-3′ (forward and reverse, respectively), 5′-VIC-ATTCCCCATTACAGCTTT-3′ and 5′-FAM-CTCACCGTAGTCCCCCAT-3′ (reference and variant allele, respectively). The Taqman genotyping reactions were carried out using Biorad’s CFX96 Touch Real-Time PCR Detection System. The utilised canine *POU1F1* reference sequences were NM_001006949.1 and NP_001006950.1 for mRNA and protein, respectively.

## Results

### Suspected pituitary dwarfism in Karelian Bear Dogs

**Fig. 1:**
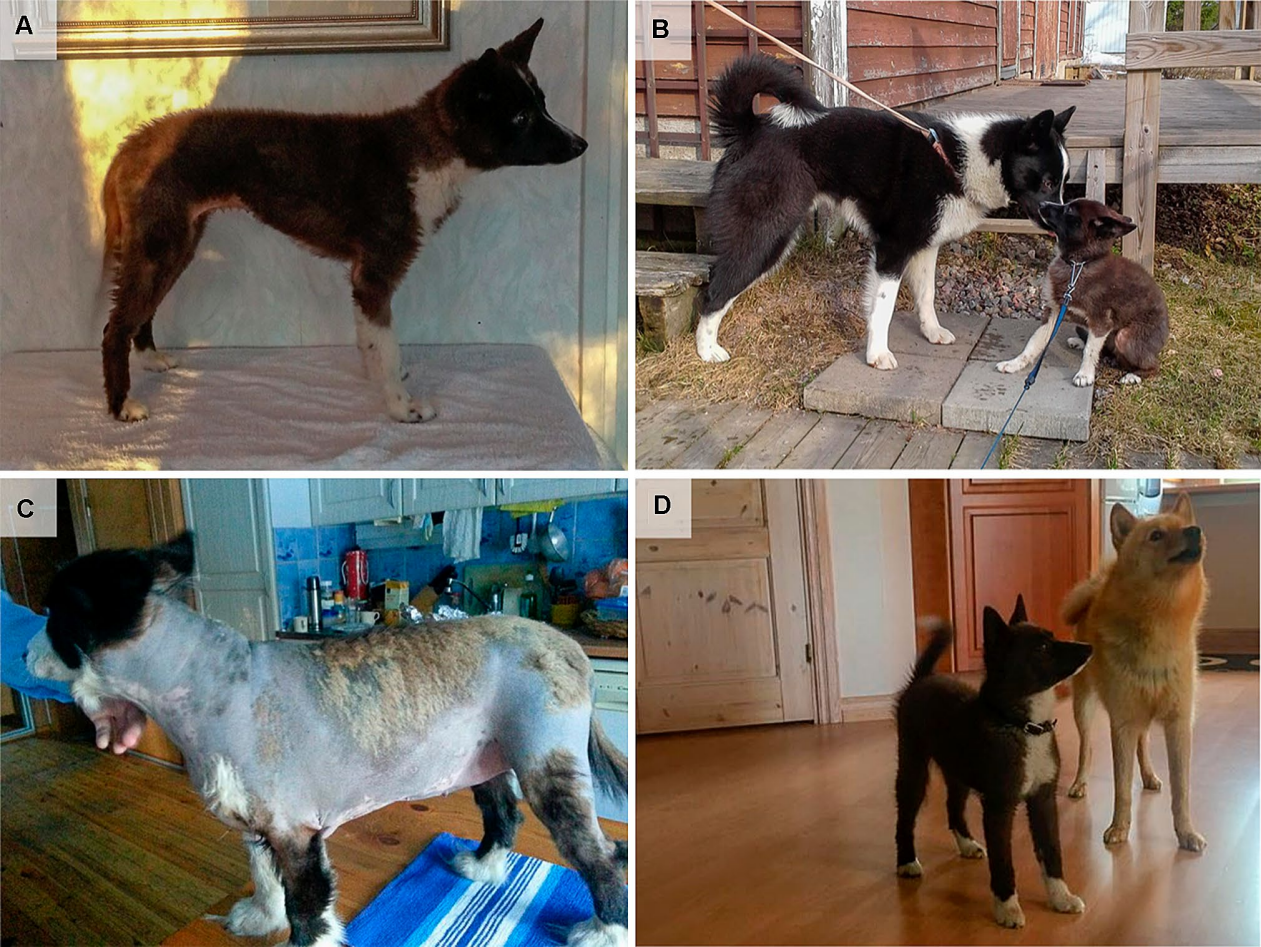
Affected KBDs. **a** An affected female KBD (case 1) at the age of 9 months. **b** An affected female dog (case 1) and an unaffected male littermate at 10 months. **c** An adult affected female KBD (case 2) showing severe hair loss. **d** An affected KBD puppy from the Swedish litter at the age of 6 months was pictured with a dog from another breed. Photos published with permission from the dog owners under the terms of the Creative Commons Attribution 4.0 International License (http://creativecommons.org/licenses/by/4.0/)

**Fig. 2:**
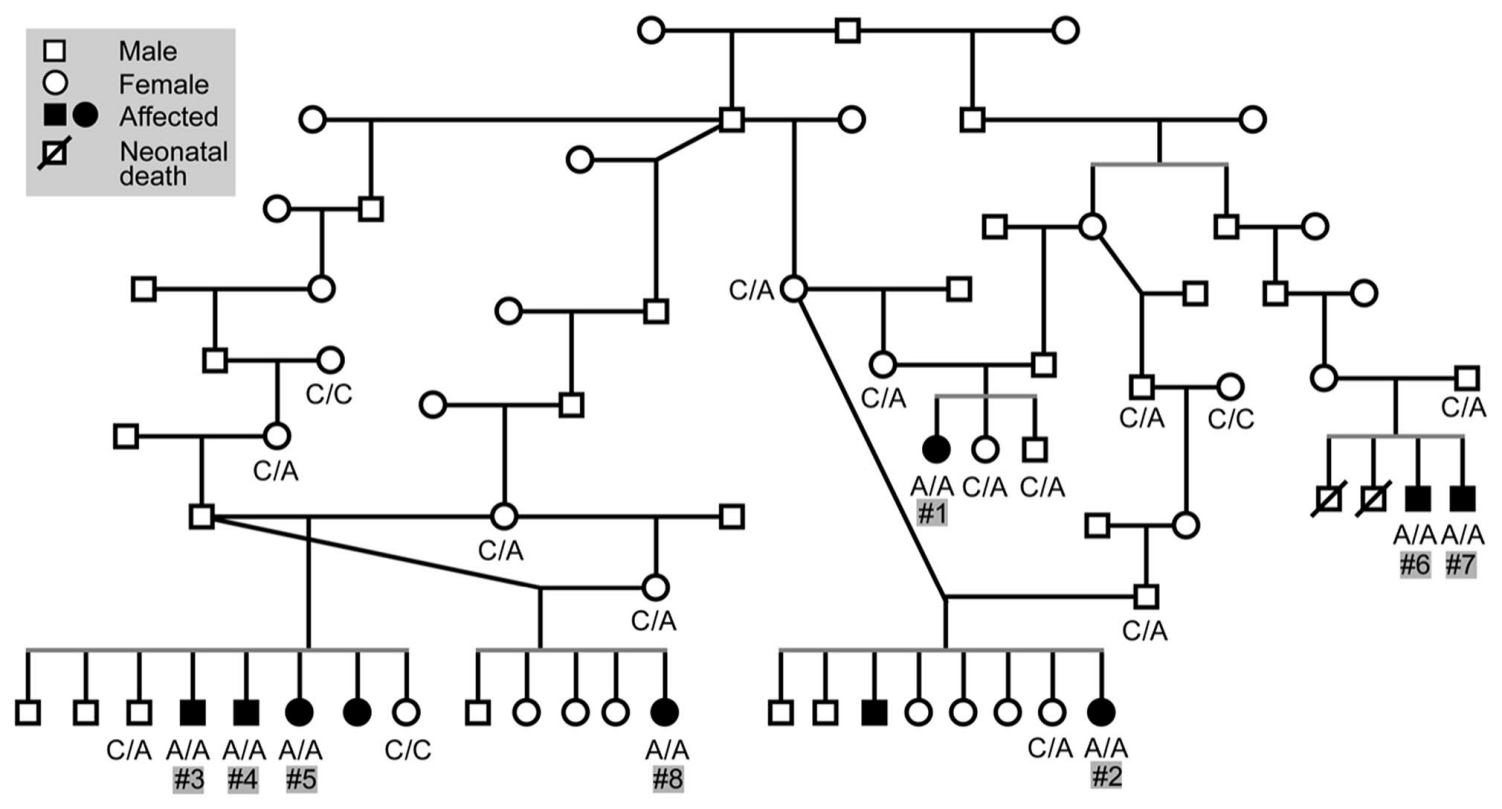
A pedigree drawn around the affected dogs suggests an autosomal recessive condition. The case numbers are denoted as #1, #2 etc. (case 1, case 2). Genotypes for the *POU1F1* c.605-3C>A are marked in the pedigree. Cases 1–5 were used in the genome-wide analysis, whereas cases 6–8 were recognised later in the variant validation phase of the study

KBD breeders with possible cases of pituitary dwarfism contacted us to initiate genetic studies. We collected clinical descriptions and blood samples from five dogs, including two Finnish and three Swedish cases. One of the Finnish dogs was a female KBD (case 1) described resembling a 6–7-week-old puppy at 13 weeks of age with a weight of 3.3 kg. At 9.5 months, the dog still weighed only 4 kg, had a “puppy-like” coat, and a height at withers of 34 cm (Fig. 1a, b). As a comparison, adult KBD females and males are typically 49–55 cm high and weigh 17–20 kg, and 54–60 cm tall and weigh 25–28 kg, respectively. A renal ultrasound had been performed for case 1 at the age of 8 weeks due to an increased blood urea nitrogen without abnormalities. Thyroid hormone levels were also within the normal limits at the age of 13 weeks. The two other suspected Finnish KBD cases, an adult female (case 2) (Fig. 1c) and male, were cousins to the mother of case 1 (Fig. 2). Owners reported them to be small with a bad puppy-like coat and hair loss. The female dog (case 2) was only 38 cm high and had substantial bilateral hair-loss affecting the trunk, neck, and proximal limbs (Fig. 1c). The skin of the dog was thin and hyperpigmented in the clinical examination with signs of inflammation. The dog had been treated for hypothyroidism. In Sweden, four suspected pituitary dwarfism cases, two females and two males were reported in a litter of eight puppies. Both of the affected males and one affected female were sampled for this study (cases 3–5) (Fig. 2). The breeder reported small size and abnormal soft coat. The affected puppies weighed 7–10 kg and were 37–40 cm high at the age of 6 months (Fig. 1d). In contrast, the unaffected littermates weighed around 20 kg and were 53–55 cm high. Serum insulin-like growth factor (S-IGF-1) level was measured to be below reference range (83 µg/l, reference 130–180 µg/l) in one affected 6-month-old Swedish dog (case 4), which supported the suspected pituitary dwarfism.

All the affected dogs were related, both sexes were affected, and parents were unaffected, suggesting an autosomal recessive mode of inheritance (Fig. 2). In addition to the suspected pituitary dwarfism cases, we collected samples from five unaffected full-siblings, four parents, three grandparents, and one great grandparent (Fig. 2).

### Disease maps to a locus in chromosome 31

**Fig. 3:**
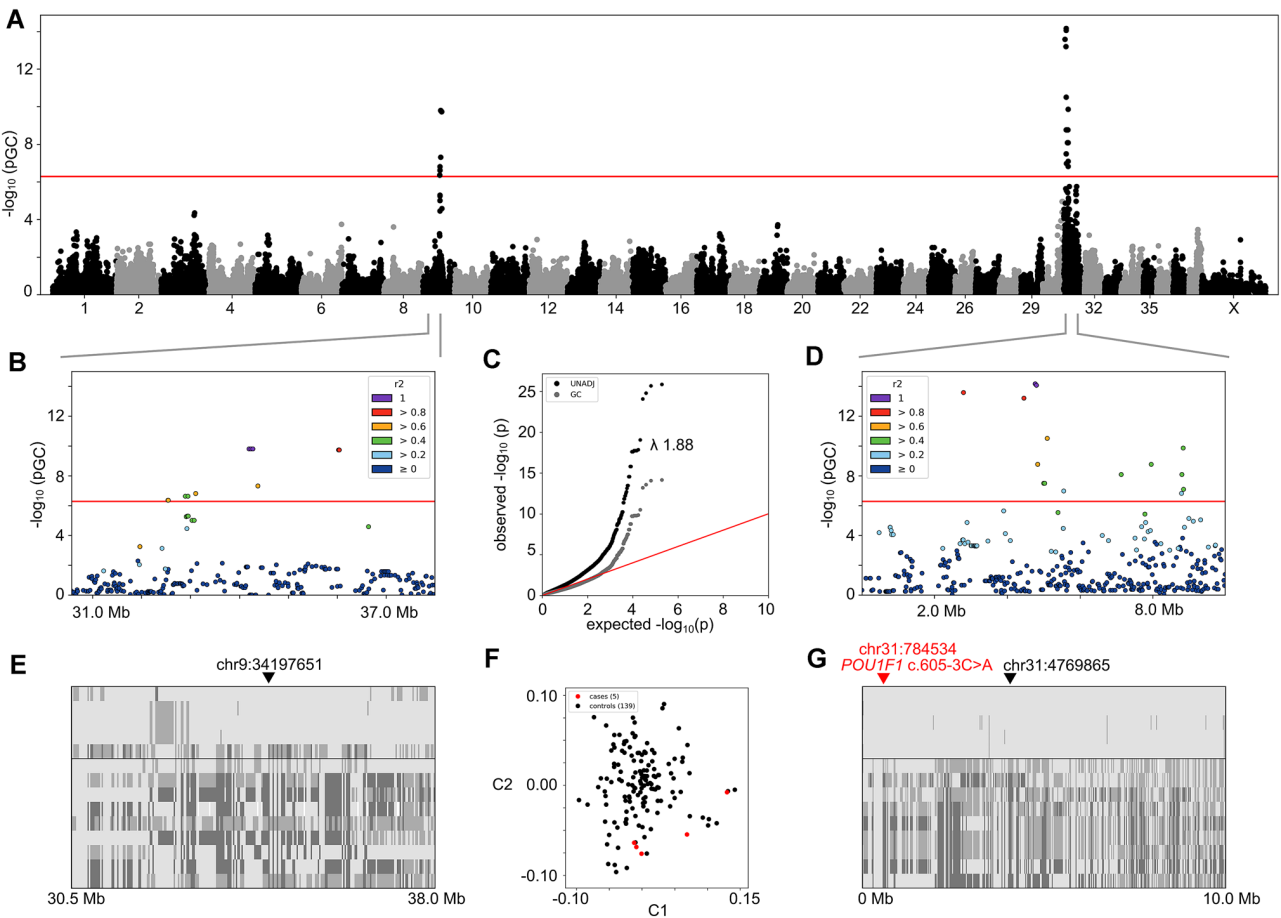
Genome-wide association study in five cases and 139 controls. SNP correlation structures (r2) in images **b** and **d** were calculated with plink. In images **e** and **g**, only ten randomly selected controls are shown for better visualisation. **a** Manhattan plot. Two genome-wide significant loci were revealed on chromosomes 9 and 31. **b** A locus plot of chr9:30–38 Mb. **c** A quantile–quantile plot of raw (UNADJ) and genomic control corrected (GC) *p* values. Lambda was 1.88 before genomic control correction. **d** A locus plot of chr31:0–10 Mb. **e** A genotype plot of chr9:30–38 Mb. One of the top SNPs at chr9:34197651 is indicated with a black triangle. All five cases are above the horizontal line, and ten randomly selected controls below the line. **f** An MDS plot with two dimensions. Cases are denoted in red and controls in black. **g** Genotype plot of chr31:0–10 Mb. The candidate variant in *POU1F1* is indicated with a red triangle and the top SNP at chr31:4769865 with a black triangle. All five cases are above the horizontal line, and ten randomly selected controls below the line

We analysed SNP array data by performing association analysis and homozygosity mapping. Case–control association analysis was carried out with the five affected KBD cases (cases 1–5, Fig. 2**)** and 139 KBD controls. The analysis revealed two loci that reached a genome-wide significance threshold of 5.19 × 10^–7^ (Fig. 3). First, fifteen markers were significant in chromosome 31 in a 6.0 Mb region at chr31:2799588–8844745 with the top SNP BICF2S23443199 at 31:4769865 (*P*_GC_ = 6.83 × 10^–15^). Second, eleven markers in chromosome 9 were significant in a 3.5 Mb region at chr9:32542006–36040587 with three top SNPs BICF2G630836109, BICF2G630836114 and BICF2G630836121 at 9:34197651 9:34236659 and 9:34275570, respectively (*P*_GC_ = 1.59 × 10^–10^). Assessment of genotypes on these two loci revealed all five cases to show extended allelic homozygosity on chromosome 31 (Fig. 3g**)**. In contrast, only four of the five cases showed homozygosity on chromosome 9 (Fig. 3b). Case 2 did not share the same homozygous block. The GWAS results were supported by homozygosity mapping, which revealed regions of homozygous allele sharing in the five affected dogs only on chromosome 31, spanning from 91,801 to 1,792,271 bp and from 4,210,204 to 8,256,918 bp.

### Whole genome sequencing uncovers a candidate splicing variant in the *POU1F1* gene

Whole genome sequencing was performed on three affected KBDs, including two Finnish (cases 1 and 2) and one Swedish dog (case 4) (Fig. 1). Single nucleotide variants and indels obtained from the affected dogs were filtered against 804 control genomes and 231 control exomes (Online material 1, Suppl Table 1) according to an autosomal recessive mode of inheritance. Since case 2 did not share the homozygous block on chromosome 9, it was not regarded as an obligate homozygote in the analysis, in case potential disease-causing variants would be found in chromosome 9. The filtering revealed altogether 321 variants, of which eighteen were not homozygous in case 2 (Online material 2, Suppl Table2). The variants were prioritised according to their position in known protein-coding genes and subsequent potential protein level impact. This approach resulted in the discovery of five exonic variants and one splicing variant (Table 1). All five exonic variants were found on chromosome 9 and comprised two synonymous changes, two missense changes, and one indel called as four separate variants by the analysis pipeline (Table 1). In silico analyses did not predict any splice effect for the synonymous changes, the missense alterations were also predicted be tolerated, and the indel variant and the missense variants to affected non-conserved amino acid positions. Furthermore, these five variants were not present in case 2 and resided in genes with no known direct association to pituitary function or disease. Therefore, they were disregarded as likely disease-causing variants. Instead, all three cases were homozygous for a possible splice variant, c.605-3C>A, located on the fourth intron of the pituitary specific transcription factor gene POU class 1 homeobox 1 (*POU1F1*), which is composed of altogether six exons. The *POU1F1* variant was heterozygous in four control samples (three KBDs and one Lapponian Herder). Variants in the *POU1F1* gene cause human CPHD (Tatsumi et al. 1992) and appeared the only suitable causative candidate variant in the affected KBDs. Alignment of the *POU1F1* variant position in multiple mammalian species revealed either cytosine or thymine nucleotide in the examined species, suggesting that adenine and guanine might not be tolerated (Online material 3, Suppl Fig. 1). Furthermore, the NNSPLICE 0.9 splice prediction tool (Reese et al. 1997) predicted this variant to weaken the splice acceptor of *POU1F1* intron 4 from a score of 0.97 to 0.67.

**Table 1:** Prioritised variants detected in NGS analyses

Chr: position	Gene	Protein	Variant	Impact	Prediction	3 NGS cases
31: 784534	*POU1F1 (NM_001006949.1)*	Pituitary-specific transcription factor	c.605-3C>A	Splice region	Weaker acceptor	3 hom
9: 31269590	*ANKFN1* (*XM_548219.6)*	Ankyrin repeat and fibronectin type III domain containing	c.729G>A, p.(Glu243=)	SYNONYMOUS	No splice effect	2 hom, 1 wt
9: 32925011	*LPO (XM_022423194.1)*	Lactoperoxidase	c.1865_1868delinsCAACCCT, p.(Glu622_Arg623delinsAlaThrLeu)	Delins	n/a	2 hom, 1 wt
9: 32934863	*MPO (XM_847352.4)*	Myeloperoxidase	c.1107C>T, p.(Ile369=)	Synonymous	No splice effect	2 hom, 1 wt
9: 32957689	*TSPOAP1 (XM_014116693.2)*	Benzodiazepine receptor-associated protein	c.3680G>C, p.(Gly1227Ala)	Missense	Neutral	2 hom, 1 wt
9: 32968794	*TSPOAP1*	Benzodiazepine receptor-associated protein	c.905C>T, p.(Ser302Leu)	Missense	Neutral	2 hom, 1 wt

We also screened a subset of the whole genome samples to identify potential disease-causing larger structural variants (SVs) and mobile element insertions (MEIs). Filtering the three KBD cases against 256 control genomes (Online material 4, Suppl Table 3) uncovered two SVs and three MEIs that were homozygous only in the affected dogs; however, all were either intronic or intergenic changes (Online material 5, Suppl Table 4). One of the three MEIs was located on chromosome 15. One was found outside of the associated region on chromosome 31. The remaining MEI and two SVs were within the associated region on chromosome 31.

### Segregation, breed specificity and the carrier frequency of the POUF1F1 variant

We next wanted to evaluate the segregation, breed-specificity, and frequency of the *POU1F1* c.605-3C > A variant in dogs. We genotyped the variant in 642 KBDs, including the five affected dogs used in the NGS and SNP chip analyses and all of their available close relatives (Fig. 1). Within the entire KBD sample cohort, 572 dogs were homozygous for the reference allele, 62 were heterozygous, and eight were homozygous for the variant allele (Table 2). The eight KBDs homozygous for the *POU1F1* variant comprised the five initially recognised cases from Finland and Sweden and three additional Finnish KBDs. These results indicated a 4% variant allele frequency and an 8% carrier frequency in the Finnish KBD population when the affected dogs and their close relatives (*n* = 23, Fig. 2) were removed from the calculation.

**Table 2:** *POU1F1* c.605-3C>A genotypes in breed cohorts

Breed	N	C/C	C/A	A/A
Karelian Bear Dog	642	572	62	8
Lapponian Herder	263	255	8	–
East Siberian Laika	50	50	–	–
West Siberian Laika	17	17	–	–
Russo-European Laika	20	20	–	–
Total	992	914	70	8

Two of the new homozygous KBDs (case 6 and 7, Fig. 2) were male puppies from a litter of four males. One puppy from the litter had died during the first day of the life, presumably having been crushed by the dam. The second puppy had stopped eating at the age of 4 days and did not gain weight even with supplementary feeding. The puppy started to have seizures and died at the age of 11 days. The third puppy appeared to develop naturally at first but was found to be blind by a veterinarian, started having movement difficulties, stopped eating and had a seizure-like occurrence. The puppy was euthanised at five weeks of age. The fourth puppy also had problems with sight and was euthanised at the age of 6 weeks. This puppy was sent for autopsy, revealing marked internal hydrocephalus for which no external cause, such as a tumour or infection, could be identified. We obtained samples from the 3rd and 4th puppies euthanised at the ages of 5 and 6 weeks and found them homozygous for the *POU1F1* c.605-3C>A variant. The sample for the dam of the litter was not available, but the sire was tested as a carrier. Of note, as the clinical presentation of these two puppies resembled a previously recognized genetic condition occurring in the breed, hypophosphatasia (Kyöstila et al. 2019), the puppies were also tested for the hypophosphatasia-associated *ALPL* gene variant. Both puppies were homozygous wildtype for the *ALPL* variant, ruling out this condition. Furthermore, in addition to these two puppies, we obtained a sample from another suspected pituitary dwarfism case (case 8, Fig. 2) from Finland, closely related to the Swedish litter. This female puppy was from a litter of five and was reported to have been of similar size to its littermates when born, but at the age of five weeks was 0.5 kg lighter than other female puppies from the litter, and also had a different coat texture (thinner and longer). This puppy was also genotyped homozygous for the *POU1F1* variant.

Since we identified one heterozygous Lapponian Herder (LH) in the NGS analysis, we decided to screen more LHs in our biobank. We genotyped 262 LHs and found seven heterozygotes for the *POU1F1* variant, but no homozygotes were identified (Table 2). The cohort of 262 LHs included 29 dogs that were specifically selected from the same breeding lines as the known heterozygous dog, whereas the rest were randomly selected from our biobank. Among the seven new heterozygous dogs, five were from the same breeding line, and two were randomly selected LHs. We also genotyped 87 Laikas from three different varieties (Table 2) to see if the *POU1F1* variant is present in a related breed. All Laikas were homozygous wildtype for the *POU1F1* variant.

Finally, we reviewed the *POU1F1* c.605-3C>A variant genotypes of 7925 dogs from 206 different breeds submitted for genetic testing at Genoscoper Laboratories but found only one heterozygous KBD in this cohort (Online material 6, Suppl table 5).

## Discussion

We discovered a homozygous *POU1F1* variant in dogs with a clinical phenotype compatible with pituitary dwarfism; a condition reported already 45 years ago in the KBD breed. The association analysis with only five closely related affected KBDs revealed two loci, on chromosome 31 and 9. The latter association did not segregate with the disease in all affected dogs and was likely due to the close relatedness of the cases. However, the locus in chromosome 31 showed complete segregation and included a known candidate gene for pituitary dwarfism, *POU1F1*, with a splice region variant likely affecting the splice acceptor in exon 4. Our results suggest that similar to humans and mice, *POU1F1* underlies pituitary dwarfism also in dogs.

POU1F1, also known as PIT-1, is a late-acting pituitary-specific transcription factor with three functional motifs: an amino-terminal transactivation domain (TAD) and two site-specific DNA binding structures POU-S and POU-H (Andersen and Rosenfeld. 1994). POU1F1 is involved in the differentiation of the somatotroph, lactotroph, and thyrotroph cell lineages and subsequent expression of the GH-1, prolactin and TSH-β genes (Fang et al. 2016). Therefore, *POU1F1* gene defects result in GH, PRL, and TSH deficiencies (Tatsumi et al. 1992; Radovick et al. 1992; Aarskog et al. 1997). In addition to CPHD, *POU1F1* has been implicated in autosomal dominant isolated GH deficiency (Sobrier et al. 2016). Clinical signs in human *POU1F1*-related CPHD include severe growth retardation in infancy, proportionate short stature, and distinctive facial features characterised by a prominent forehead, midface hypoplasia, deep-set eyes depressed nasal bridge. Clinical manifestations are present from early life and are associated with a normal or hypoplastic anterior pituitary, while TSH deficiency can be more variable (Radovick et al. 1992; Aarskog et al. 1997; Turton et al. 2005; Fang et al. 2016). The major clinical characteristics in canine pituitary dwarfism are proportionate small stature and an abnormal coat. Low somatomedin values were previously reported in two affected KBDs (Andresen and Willeberg. 1976). In the affected KBDs included in the current study, low serum IGF-1 value had been measured in one dog (case 4), whereas one dog was diagnosed with hypothyroidism (case 2), and one was reported to have normal thyroid hormone values at the age of 13 weeks (case 1).

At least 30 different disease-causing *POU1F1* variants have been described to date in humans, of which few are dominant, with the majority showing recessive inheritance (Fang et al. 2016). Our results confirmed autosomal recessive inheritance in the affected KBDs. The *POU1F1* splice region variant in affected dogs was predicted to affect splicing. Although functional studies are necessary to confirm the variant's potential splicing effect, it is possible that the variant could result in abnormal splicing, which could, for instance, truncate the C-terminal end of the encoded polypeptide. Unfortunately, we did not have access to the pituitary tissue from affected dogs to test the stability of the mutated transcript and to prove the pathogenicity of the variant. This remains an essential future effort when affected samples become available. Furthermore, the pathogenic effects of the identified variant could be examined through an exon trapping assay. However, there was variation in the clinical presentations of the affected dogs, which could be due to some leakage of the wild-type allele, typical for splice site variants (Hytönen et al. 2016).

We identified the *POU1F1* splice region variant in a homozygous state in altogether eight KBDs. The phenotype of six affected dogs (cases 1–5 and 8) was compatible with the clinical description provided by Andresen and Willeberg (1976). Simultaneously, two homozygous male puppies had been euthanised at 5 and 6 weeks of age due to failure to thrive and blindness, suggesting variation in the clinical phenotype. These two puppies also had littermates that died soon after birth without being screened for the *POU1F1* variant, and therefore, it is unsure whether they may have also been homozygotes. In German Shephard CPHD, several affected animals were reported to have been intensively nursed and hand-reared during the first three weeks of life due to failure to thrive, which indicated that canine hypopituitarism might go in some instances undetected because of postnatal failure to thrive (Allan et al. 1978).

Despite limitations to validate some of our genetic findings due to lack of appropriate tissue-specific patient material, our study provides compelling evidence to confirm the suspected recessive pituitary dwarfism in KBD, providing a novel comparative large animal model for human CPHD. Simultaneously, the genetic test devised based on this study will have practical implications for improving veterinary diagnostics and breeding plans for better care and eradicating the condition from the affected breeds.

## Supplementary Information

Below is the link to the electronic supplementary material.

Supplementary file1 List of 804 control genomes and 231 control exomes used in the study (XLSX 13 KB)

Supplementary file2 List of case-specific variants in the sequence analysis (XLSX 39 KB)

Supplementary file3 Alignment of the POU1F1 variant position in multiple mammalian species (DOCX 21 KB)

Supplementary file4 List of 256 control genomes used in the SV and MEI filtering (XLSX 10 KB)

Supplementary file5 Summary of the identified case-specific intronic or intergenic SVs and MEIs (XLSX 9 KB)

Supplementary file6 Summary of POU1F1 variant screening in 7925 dogs from 206 different breeds (XLSX 30 KB)

## Data Availability

Whole-genome sequencing data from three dogs have been submitted to the Sequence Read Archive in BAM format with BioProject accession number PRJNA684429 (https://www.ncbi.nlm.nih.gov/bioproject/PRJNA684429/) and BioSample accession numbers SAMN17057666, SAMN17057667, and SAMN17057668.
