# Supplementary material for: Intronic variant in POU1F1 associated with canine pituitary dwarfism
Source: Hum Genet. 2021 Feb 6;140(11):1553–62. doi: 10.1007/s00439-021-02259-2 (PMC8519942; doi:10.1007/s00439-021-02259-2)
Supplement: Supplementary file 3 — Supplementary file3 Alignment of the POU1F1 variant position in multiple mammalian species (DOCX 21 KB) [file 439_2021_2259_MOESM3_ESM.docx]

Dog *C.familiaris, wt* GAGGAAGCTGAGCAAGTGGGAGgtactgaagttgtcg...tattccccattacagCTTTATACAATGAAAAAGTGGGA

Dog *C. familiaris, mut* GAGGAAGCTGAGCAAGTGGGAGgtactgaagttgtcg...tattccccattaaagCTTTATACAATGAAAAAGTGGGA

Human *H. sapiens* GAGGAAGCTGAGCAAGTAGGAGgtactaaattgtgtc...ttttccacattatagCTTTGTACAATGAAAAAGTGGGA

Chimp *P. troglodytes* GAGGAAGCTGAGCAAGTAGGAGgtactaaattgtgtc...ttttccacattatagCTTTGTACAATGAAAAAGTGGGA

Rhesus macaque *M. mulatta* GAGGAAGCTGAGCAAGTAGGAGgtactaaactgtgtc...ttttccacattatagCTTTGTACAATGAAAAAGTGGGA

Marmoset *C. jacchus* GAGGAAGCTGAGCAAGTGGGAGgtactgaactgtctc...tattccaccttatagCTTTGTACAATGAAAAAGTGGGA

Galago *O. garnettii* GACGAGGCGGAGCAAGTAGGAGgtactaatgctggat...gtttccatactatagCGTTGTACAATGAAAAAGTGGGA

Mouse *M. musculus* GAGGAAGCTGAGCAGGTCGGAGgtactgaagccaatc...ctttctacattccagCTTTGTACAATGAGAAGGTGGGA

Rat *R. norvegicus* GAGGAAGCTGAGCAGGTCGGAGgtactgaagcacctt...ctttccacatttcagCTTTGTACAATGAAAAAGTGGGA

Naked mole rat *H. glaber* GAGGAAGCCGAGCAAGTAGGAGgtactaactagactg...ttttccacattccagCTTTGTACAATGAAAAAGTGGGA

Hedgehog *E. europaeus* GAGGAAGCTGAGCAAGTGGGAGgtattcaaactgttc...ttggccccattacagCTTTATACAGTGAAAAAGTGGGA

Pig *S. scrofa* GAGGAAGCTGAGCAAGTAGGAGgtactaaagctgtgc...ttttccccatcacagCTTTATACAATGAGAAAGTGGGA

Cow *B. taurus* GAGGAAGCCGAGCAAGTAGGAGgtacaaaagctgtgt...gttttcgcatcacagCTTTATACAATGAGAAAGTTGGT

Dolphin *T. truncatus* GAGGAAGCCGAGCAAGTAGGAGgtactaaagttgttt...ttttccccatcacagCTTTATACAATGAGAAAGTGGGT

Horse *E. caballus* GAGGAGGCTGAGCAAGTAGGAGgtagccaagcgctat...ttttctccattgcagCTTTGTACAATGAAAAAGTGGGA

Panda *A. melanoleuca* GAGGAAGCTGAGCAAGTGGGAGgtactgaagttacag...ttttccccattacagCTTTATACAATGAAAAAGTAGGA

Cat *F. catus* GAGGAAGCAGAGCAAGTCGGAGgtactgaatttagat...ttttccccattacagCTTTATACAATGAAAAAGTGGGA

Elephant *L. africana* GAGGAAGCTGAGCAAGTAGGAGgtacgaaagctatgt...ttttccccattacagCATTATACAATGAAAAAGTGGGA

Opossum *M. domestica* GATGAAGCTGAGCAAGTAGGAGgtaccatagctacat...ttttcataaatatagCTTTATACAATGATAAAGTGGGG

Platypus *O. anatinus* GATGAAGCAGAACAAGTTGGAGgtatagattgatggt...cttttttaaaaacagCTTTGTACAATGAAAAAGTTGGG

** ** ** ** ** ** ******* *** ** **** *** ** ** **

**Supplementary Figure 1.** Alignment of the *POU1F1* c.605-3C>A variant position in multiple mammalian species. An arrow denotes the variant position in the fourth intron of the *POU1F1* gene. Capital letters indicate exons.

Used sequence references:

>NM_001006949.1 Canis lupus familiaris POU class 1 homeobox 1 (POU1F1), mRNA

>NM_000306.4 Homo sapiens POU class 1 homeobox 1 (POU1F1), transcript variant alpha, mRNA

>XM_003318202.4 PREDICTED: Pan troglodytes POU class 1 homeobox 1 (POU1F1), transcript variant X1, mRNA

>NM_174579.4 Bos taurus POU class 1 homeobox 1 (POU1F1), mRNA

>NM_008849.6 Mus musculus POU domain, class 1, transcription factor 1 (Pou1f1), transcript variant 1, mRNA

>NM_013008.3 Rattus norvegicus POU class 1 homeobox 1 (Pou1f1), mRNA

>XM_023238868.1 PREDICTED: Felis catus POU class 1 homeobox 1 (POU1F1), mRNA

>XM_004321204.2 PREDICTED: Tursiops truncatus POU class 1 homeobox 1 (POU1F1), transcript variant X1, mRNA

>XM_019802253.1 PREDICTED: Ailuropoda melanoleuca POU class 1 homeobox 1 (POU1F1), mRNA

>XM_002761292.2 PREDICTED: Callithrix jacchus POU class 1 homeobox 1 (POU1F1), mRNA

>XM_007500749.1 PREDICTED: Monodelphis domestica POU class 1 homeobox 1 (POU1F1), mRNA

>XM_023548171.1 PREDICTED: Loxodonta africana POU class 1 homeobox 1 (POU1F1), mRNA

>XM_007530716.1 PREDICTED: Erinaceus europaeus POU class 1 homeobox 1 (POU1F1), transcript variant X1, mRNA

>XM_004857908.3 PREDICTED: Heterocephalus glaber POU class 1 homeobox 1 (Pou1f1), transcript variant X1, mRNA

>NM_214163.1 Sus scrofa POU class 1 homeobox 1 (POU1F1), mRNA

>XM_001501258.3 PREDICTED: Equus caballus POU class 1 homeobox 1 (POU1F1), mRNA

>XM_003430572.3 PREDICTED: Ornithorhynchus anatinus POU class 1 homeobox 1 (POU1F1), transcript variant X1, mRNA

>NM_001042860.1 Macaca mulatta POU class 1 homeobox 1 (POU1F1), mRNA

>XM_012801585.1 PREDICTED: Otolemur garnettii POU class 1 homeobox 1 (POU1F1), transcript variant X1, mRNA
